# Understanding MMPI-2 response structure between schizophrenia and healthy individuals

**DOI:** 10.3389/fpsyt.2022.918999

**Published:** 2022-07-28

**Authors:** Yu Cheng Hsu, Zhiyu Ye, Lisha Dai, Yaqin Jing, Kwok-Leung Tsui, Paul S. F. Yip, Wentian Li, Qingpeng Zhang

**Affiliations:** ^1^School of Data Science, City University of Hong Kong, Kowloon, Hong Kong SAR, China; ^2^School of Education Research, China University of Geosciences, Wuhan, China; ^3^Research Center for Psychological and Health Sciences, China University of Geosciences, Wuhan, China; ^4^Wuhan Mental Health Center, Wuhan, China; ^5^Tongji Medical College, Huazhong University of Science and Technology, Wuhan, China; ^6^Grado Department of Industrial and System Engineering, Virginia Polytechnic Institute and State University, Blacksburg, VT, United States; ^7^Department of Social Work and Social Administration, The University of Hong Kong, Kowloon, Hong Kong SAR, China

**Keywords:** schizophrenia, MMPI-2, Bayesian network, personality assessment, network classification

## Abstract

**Background:**

Using Minnesota Multiphasic Personality Inventory-2 (MMPI-2) clinical scales to evaluate clinical symptoms in schizophrenia is a well-studied topic. Nonetheless, research focuses less on how these clinical scales interact with each other.

**Aims:**

Investigates the network structure and interaction of the MMPI-2 clinical scales between healthy individuals and patients with schizophrenia through the Bayesian network.

**Method:**

Data was collected from Wuhan Psychiatric Hospital from March 2008 to May 2018. A total of 714 patients with schizophrenia and 714 healthy subjects were identified through propensity score matching according to the criteria of the International Classification of Diseases (ICD-11). Separated MMPI-2 clinical scales Bayesian networks were built for healthy subjects and patients with schizophrenia, respectively.

**Results:**

The Bayesian network showed that the lower 7 scale was a consequence of the correlation between the lower 2 scale and the greater 8 scale. A solely lower 7 scale does yield neither a lower 2 scale nor a higher 8 scale. The proposed method showed 72% of accuracy with 78% area under the ROC curve (AUC), similar to the previous studies.

**Limitations:**

The proposed method simplified the continuous Bayesian network to predict binary outcomes, including other categorical data is not explored. Besides, the participants might only represent an endemic as they come from a single hospital.

**Conclusion:**

This study identified MMPI-2 clinical scales correlation and built separated Bayesian networks to investigate the difference between patients with schizophrenia and healthy people. These differences may contribute to a better understanding of the clinical symptoms of schizophrenia and provide medical professionals with new perspectives for diagnosis.

## Introduction

Schizophrenia is a major psychiatric disorder diagnosed by observing a combination of symptoms (1). Schizophrenia is the most common group of psychiatric disorders characterized by basic personality changes, splitting of thinking, emotions, and behavior, and incompatibility of mental activities with the environment, and the MMPI-2 is a recognized measurement tool to assist in screening patients with schizophrenia. The lifetime prevalence in urban China areas is 0.83%, and it was estimated that there were 7.16 million people in China suffering from schizophrenia in 2010 (2). Diagnosing schizophrenia is the first step for professionals to take intervention and provide them suitable support. Early detection and diagnosis of schizophrenia are of research interest because they can benefit both clinical professionals and patients. Early detection and filtering without clinical professional effort can relieve the burden in the clinics and facilitate the early intervention, which alleviates the severity and chronicity (3).

Diagnosis of schizophrenia in clinical fields is difficult, and current practices have received several challenges. The major reason is that diagnosing schizophrenia in clinics relies on clinical observations of specific symptoms over a month using the Fifth Edition of the Diagnostic and Statistical Manual of Mental Disorders (DSM-5) (1) and the International Classification of Disease (ICD) (4) guidelines. Though these guidelines are widely adopted in clinics, they have been criticized in several aspects. 1. A high diagnostic threshold is needed for schizophrenia (5). 2. Various symptom profiles occur in the exiting guideline (6, 7). These difficulties make the diagnosis of schizophrenia time-consuming and heavily rely on professional clinical experience. It is hard for professionals to diagnose accurately and effectively. An innovative exploration of clinical symptoms of schizophrenia is conducive to helping medical professionals better understand schizophrenia, and facilitate the realization of early detection and diagnosis.

Existing research studies on detecting schizophrenia in the early stage can be categorized into using magnetic resonance image (MRI) (8–10) and the psychometric test (11–13), such as the Minnesota Multiphasic Personality Inventory (MMPI). The psychometric test is a self-completed questionnaire that is not limited by the availability of MRI scanners and radiologists. The psychometric test provides an off-the-shelf and less expensive option than MRI for early scanning and detection of schizophrenia.

Many studies have examined the feasibility of differentiating schizophrenia patients from other psychiatric disorders or healthy adults using MMPI. Using the available clinical scales as indicators of schizophrenia is a common approach (11–13), and the other approach (14) is constructing a new scale by selecting relevant questions from the MMPI questions. Building a new scale can effectively screen schizophrenia patients by selecting relevant questions in MMPI tests. The shortcoming is that these specific scales were not widely adopted in other circumstances and, therefore, adoption in different demographics might require additional validation before use. In contrast, using existing scales might not be as discriminative as specific scales, but it is convenient and widely used in most demographics.

Early research studies in the MMPI have studied the discriminative power of MMPI Clinical scales and the difference between schizophrenia patients and other populations. Many research studies (11–13, 15) indicated a statistical significance of a higher Scale 8 in schizophrenia patients. Research study (16) pointed out that patients with schizophrenia usually exhibit lower scores on Scale 2 (Depression). Evidence (17) also supported the aforementioned finding in the MMPI-2, a revised version of MMPI. In summary, different from the 278 codetype in the MMPI-2 manual, most of the research has a major consensus (11, 12, 15, 18) is that a lower score in Scale 2 (Depression), and 7 (Psychasthenia) and a higher score in Scale 8 (Schizophrenia) are a sign of schizophrenia.

Nonetheless, research teams less addressed the correlations between MMPI clinical scales in different populations explicitly. A previous study (19) pointed out a high correlation between clinical Scale 8 and validity Scale F. The existence of correlation among MMPI-2 scales affects the interpretation of classification results because such correlation within MMPI-2 clinical scales violates the assumption of the regression that these independent variables are independent. This research investigates these correlations between MMPI-2 clinical scales and examines whether there is any difference in MMPI-2 clinical scales correlation between schizophrenia patients and healthy individuals, which has an important reference value for the interpretation of the scale results and the clinical work of diagnosis of schizophrenia.

This study used a data-driven approach and investigated the correlation and its difference in the MMPI-2 clinical scales between schizophrenia patients and healthy individuals through the separated Bayesian network. This study examined the interaction between Scale 278 in patients with schizophrenia, providing a more comprehensive understanding and sketching of schizophrenia MMPI-2 clinical scales. Overall, the finding could benefit medical professionals in better understanding the symptoms of schizophrenia and providing guidance for clinical diagnosis.

## Materials and methods

### Participants

A total of 9,216 participants completed MMPI-2 during their visit to Wuhan Psychiatric Hospital from March 2008 to May 2018. Their admission-related ICD diagnosis and demographics were also collected at the time of conducting MMPI-2. Diagnoses for all patients with schizophrenia were based on the Structured Clinical Interview for the Diagnostic and Statistical Manual of Mental Disorders (SCID-I/P) (20). A panel of research psychiatrists and psychologists re-evaluated clinical data using ICD-9 criteria and made a final consensus diagnosis. Their diagnosis is healthy (ICD-9:0.0, *n* = 3,135), other unspecified complications of medical care not elsewhere specified (ICD-9: 999.9, *n* = 5,168), schizophrenia (ICD-9: 295.9, *n* = 714), and other psychiatric disorders (*n* = 199). The diagnosis was made according to ICD guidelines by psychiatric doctors.

A huge portion of healthy participants in the psychiatric hospital come from the fact that some enterprises or community organizations might entrust psychiatric hospitals with the mental health examination before employment. These healthy people are helpful for us in comparing the MMPI-2 clinical scale difference between them and those with schizophrenia.

#### Ethics

The study was conducted in accordance with the Declaration of Helsinki (as revised in 2013). Before the survey, the Institutional Review Board of Wuhan Mental Health Center approved the survey protocol and the above-mentioned procedures of informed consent (No.: KY2021.08.05). Participants' written informed consent was obtained as well.

### Measure instruments and procedure

The final sample used in this study consists of 714 patients with schizophrenia, and the other 714 healthy participants were included through propensity score matching using MatchIt (21) package using the optimal full matching method (22) in R. Among 3,849 participants included for the propensity score matching, 3,553 of them used 200 items MMPI-2, 257 of them used 399 items MMPI-2 and the rest of 39 participants used 566 items MMPI-2. Propensity score matching based on age, gender, educational level, and marital status was applied to minimize the demographic difference in these aspects and reduce the bias and confounding factors from demographics. The detailed decomposition of the participants is tabularized in Table 1.

**Table 1:** Demographics of participants included in this study.

	**Overall**	**Healthy**	**Schizophrenia**
n	1,428	714	714
Age (year)	27.82 (12.11)	28.18 (12.75)	27.47 (11.42)
Gender (1:Male, 0:Female)	0.75 (0.43)	0.74 (0.44)	0.76 (0.43)
Education level (Year of receiving education)	11.39 (3.41)	11.33 (3.61)	11.45 (3.20)
Marital status (1:married, 0:others)	0.30 (0.66)	0.31 (0.59)	0.30 (0.73)
MMPI-2 L, F, K and clinical scales			
L scale	53.96 (14.91)	50.19 (13.43)	57.74 (15.37)
F scale	59.18 (14.88)	55.65 (14.38)	62.70 (14.55)
K scale	53.92 (14.04)	50.96 (13.01)	56.89 (14.41)
Scale 1 Hypochondriasis (Hs)	58.72 (12.80)	58.17 (14.10)	59.26 (11.13)
Scale 2 Depression (D)	55.03 (13.39)	56.11 (15.62)	53.95 (10.61)
Scale 3 Hysteria (Hy)	61.68 (14.39)	60.38 (14.42)	62.99 (14.25)
Scale 4 Psychopathic Deviate (Pd)	56.99 (12.99)	57.11 (14.53)	56.87 (11.24)
Scale 5 Masculinity/Femininity (MF)	51.99 (12.05)	51.19 (13.51)	52.78 (10.34)
Scale 6 Paranoia (Pa)	54.38 (10.36)	52.54 (10.55)	56.23 (9.85)
Scale 7 Psychasthenia (Pt)	55.91 (12.14)	57.81 (13.58)	54.00 (10.16)
Scale 8 Schizophrenia (Sc)	56.99 (12.63)	56.19 (13.31)	57.79 (11.88)
Scale 9 Hypomania (Ma)	55.55 (10.54)	54.66 (11.01)	56.44 (9.98)
Scale 0 Social Introversion (Si)	45.08 (11.06)	47.06 (12.54)	43.10 (8.93)

### Data analysis

#### Bayesian network model

The Bayesian network method is an adequate method to model the MMPI-2 scales and their relation to schizophrenia, as researchers are concerned about how the combination of different scales affects the prediction outcome. The Bayesian network provides the fruitful interpretation of the dependency structure in the MMPI-2 clinical scales and, thus, provides both the prediction outcome and insightful interpretations.

Bayesian networks were used to analyze the MMPI-2 clinical scales and three validity scales, L, F, and K, in this study. Bayesian network is a directed acyclic graphical (DAG) model. Bayesian networks denote the variables (i.e., the MMPI clinical scales in this study) as the node. While a node pair shows a dependence structure, then the arc is connected between them. If a node is connected by several nodes, that means this node is jointly dependent on these nodes. The arc strength is to quantify the magnitude of the dependence structure. The measurement criterium is Bayesian information criteria that measure the model's goodness.

If a normal distributed variable node *Y* is pointed by a normally distributed nodes set *X* then there is a linear probabilistic relationship. In other words, it can be expressed as *Y* = β*x*+*c*, where β is the coefficient and *c* is the constant. The Bayesian network quantifies the linear correlation between each MMPI-2 and delineates the interaction of these variables. Furthermore, it examines the difference in correlation between schizophrenia patients and healthy people. The correlation test was made the method proposed by Wang et al. (23), which performs the hypothesis test in a non-parametric manner.

Dependency structures in Bayesian networks usually rely on domain knowledge to determine its existence and direction. Since domain knowledge might not always be available and comprehensive, this study used structure learning algorithms to provide a statistical solution to construct the Bayesian network without domain knowledge. The presented research first learned separated Bayesian network structures for healthy individuals and patients with schizophrenia on MMPI-2 scales (10 clinical scales, L, F, and K scale) through H2PC (24) method. After the structures were learned, the parameters of each arc were fitted by maximizing the likelihood. Bayesian network structure learning and fitting were implemented using the bnlearn (25) package in R.

The classification assumes that the new observation would exhibit a higher probability in the corresponding Bayesian networks if they are in the same diagnosis group. This assumption is valid when each group's portion is a fixed constant using the Bayes rule shown in Equation 1.


(1) $$\begin{array}{r} \frac{p\left(X=x|Y=Normal\right)}{p\left(X=x|Y=Schizophrenia\right)}\propto \frac{p\left(Y=Normal|X=x\right)}{p\left(Y=Schizophrenia|X=x\right)} \end{array}$$

The portion of the patients with schizophrenia and healthy people population is controlled by the propensity matching in this study. In other words, the odd of the new observation in the healthy participants and schizophrenia patients' MMPI-2 response is proportional to the probability that the new observation occurs in the corresponding Consequently, the portion can be a helpful indicator in differentiating healthy participants and patients with schizophrenia.

#### Evaluation metrics

Accuracy, area under the curve (AUC), and F1 score are used as an indicator of the classification performance. AUC is the area under the receiver operating curve (ROC), which is the plot of true positive (TP) and false positive (FP) at different thresholds. F1 score is the harmonic mean of precision and recall, which measure the accuracy with consideration of both positive cases and negative cases.

## Results

Compared with the logistic regression, the Bayesian network performed slightly better in terms of accuracy, AUC, and F1 score. 100-iteration random-shuffle-split cross-validation (100-RSSCV) was used to test the proposed method. In each iteration, 80% of the samples were randomly selected to construct the model, and the rest of the 20% were for testing the model. The proposed method was compared with logistic regression using only clinical scales 2, 7, and 8, which were frequently reported in the previous studies. Table 2 displays the prediction performance of each method. The proposed method performed the best among the rest of the two baselines in terms of all evaluation metrics, including accuracy, F1 score, and area under the curve (AUC).

**Table 2:** Results of the proposed method and other indicators used in the previous studies.

	**Accuracy**	**F1**	**AUC**
Proposed method	0.72 (0.02)	0.75 (0.02)	0.78 (0.03)
Clinical Scale 278	0.71 (0.02)	0.71 (0.02)	0.71 (0.03)

*The bold effect used to indicate the best highest among the column*.

### Common features among patients with schizophrenia and healthy participants

**Figure 1:**
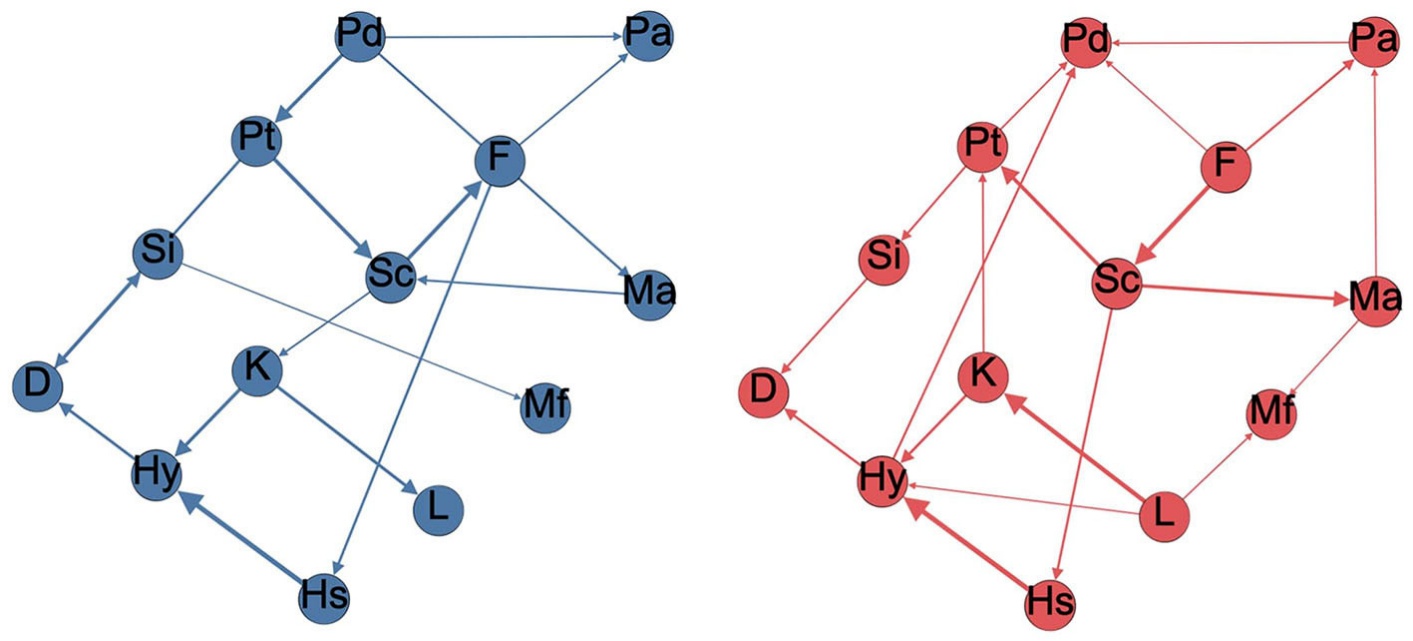
Minnesota Multiphasic Personality Inventory (MMPI)-2 scales Bayesian network for healthy participants (left) and patients with schizophrenia (right). The thickness of the arcs is proportional to the BIC value, D, Depression; F Infrequency; Hs, Hypochondriasis; Hy, Hysteria; K, Defensiveness; L, Lie; Ma, Hypomania; Mf, Masculine feminine; Pa, Paranoia; Pd, Psychopathic deviate; Pt, Psychasthenia; Sc, Schizophrenia; Si, Social Introversion.

Figure 1 displayed the developed Bayesian network for healthy participants and patients with schizophrenia using H2PC structure learning using the whole datasets. The thickness of the arcs in Figure 1 was scaled according to the arc strength measured by Bayesian Information Criterion (BIC).

The Bayesian network in the MMPI-2 response has revealed that healthy participants tended to have simpler network structures than patients with schizophrenia. Healthy participants' Bayesian network on MMPI-2 response had 16 arcs. In contrast, the Bayesian network of patients with schizophrenia on MMPI-2 response had 21 arcs. Those extra edges slightly overall goodness of fit of the Bayesian network model (slight change in BIC).

There were some common arcs that existed in both schizophrenia patients' networks and healthy participants' networks. L- K, K-Hy, Hs- Hy, Sc-Pt, F-Sc, Si-D, and Pd-Pt arcs existed in both networks with high arc strengths. This phenomenon implied that these scales were dependent on each other, neglecting the effect of schizophrenia.

### Difference of Bayesian network between patients with schizophrenia and healthy participants

**Figure 2:**
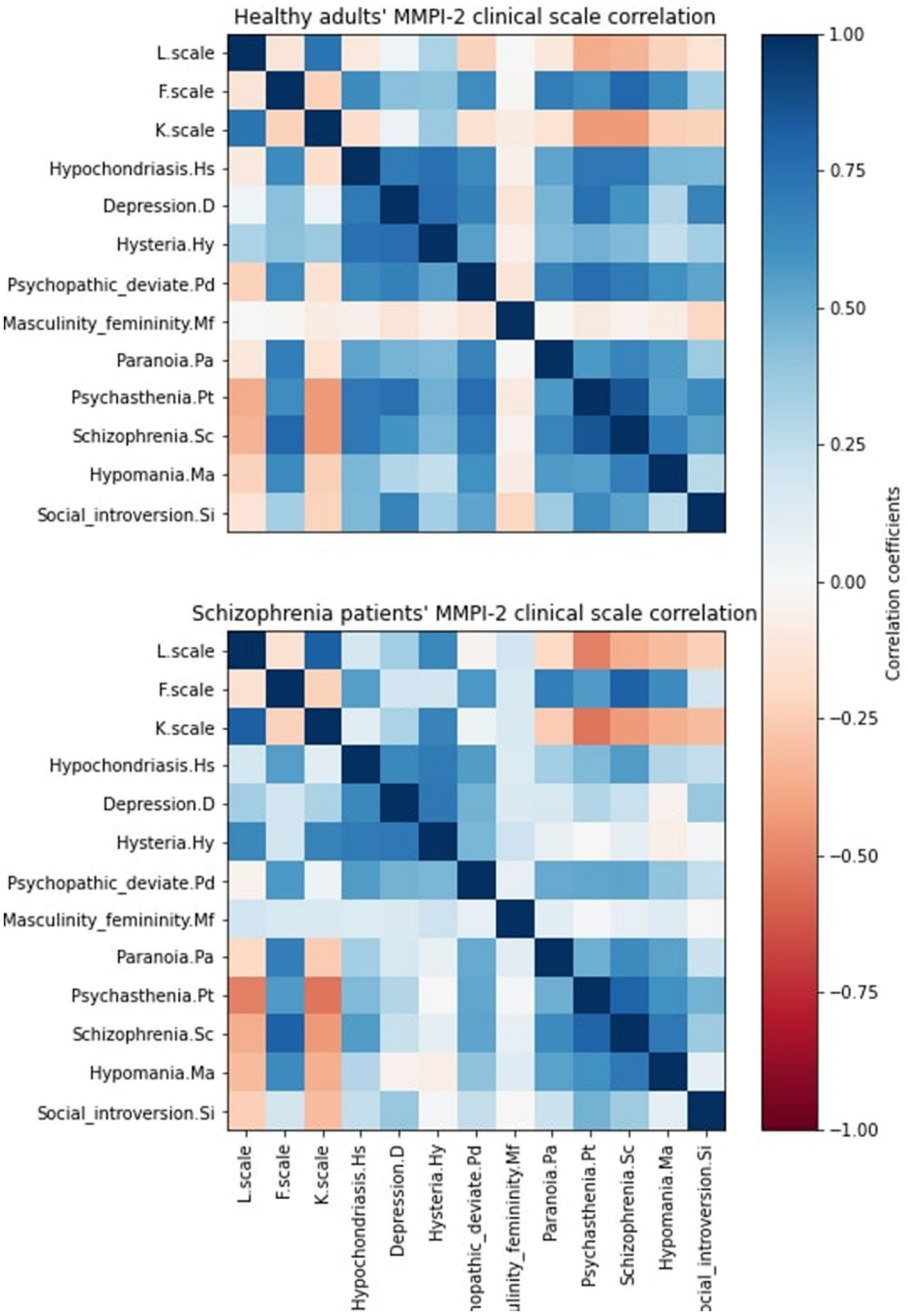
MMPI-2 correlation matrix of healthy adults (top) and patients with schizophrenia (bottom). The correlation coefficient is calculated by Pearson correlation coefficients.

Figure 2 has shown the correlation between MMPI response in healthy participants and patients with schizophrenia. The Bayesian network could quantify the linear relationships among the MMPI-2 clinical scales and make the decision. Healthy people exhibited a strong correlation between Pt and D scales (Pearson correlation = 0.74) and Pd and Ma scales (Pearson correlation = 0.61). In contrast, patients with schizophrenia have revealed a lower correlation for Pt and D scales (Pearson correlation = 0.29) and Pd and Ma scales (Pearson correlation = 0.41). Besides, both patients with schizophrenia and healthy participants had a moderate correlation coefficient between the F and Hs scales (Pearson correlation = 0.55, 0.63, respectively).

**Figure 3:**
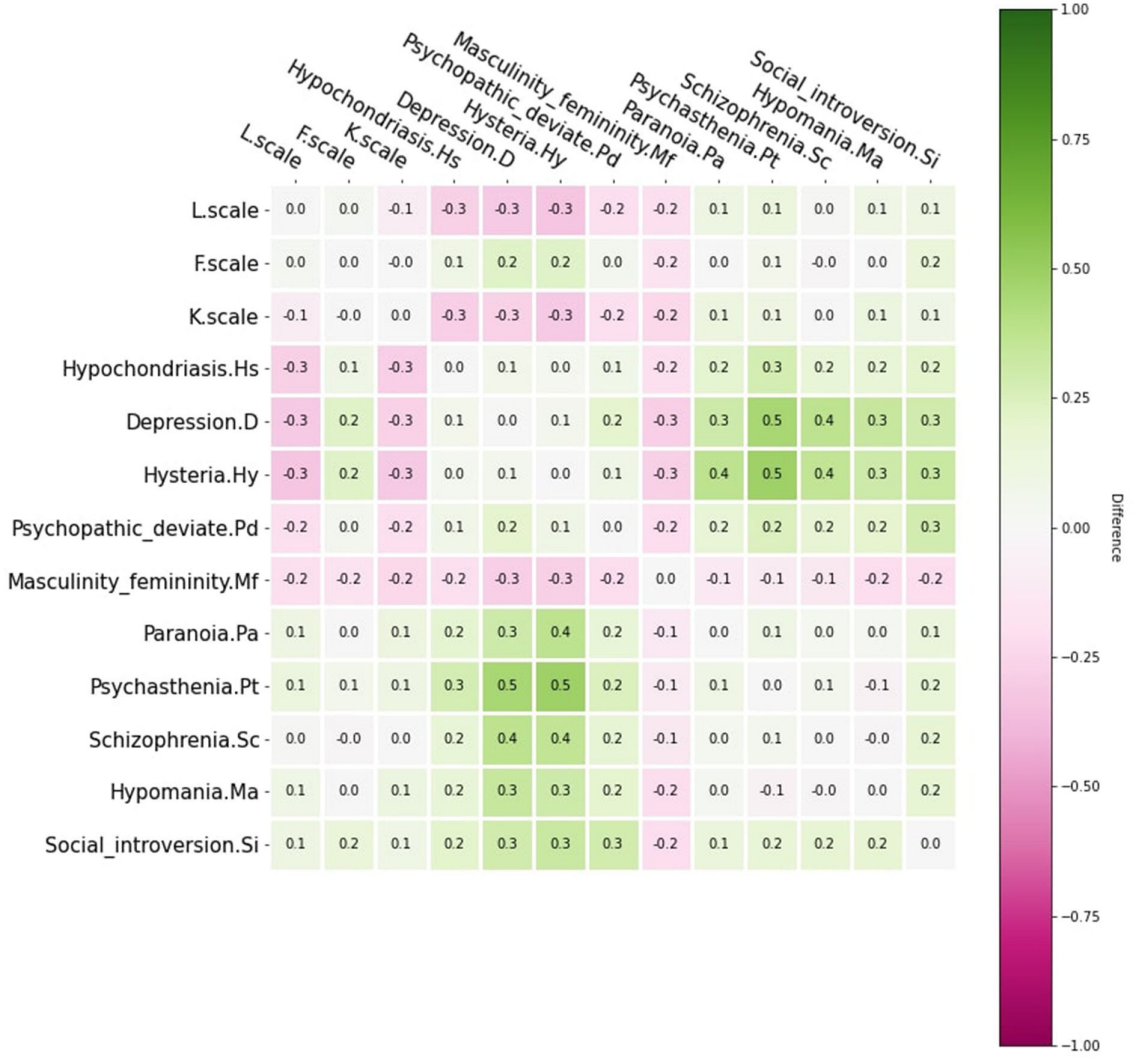
MMPI-2 scales correlation matrix difference between healthy people and patients with schizophrenia.

Figure 3 illustrates their correlation difference using the Pearson correlation coefficient. The correlation difference of MMPI response majorly occurred at the Hs, D, Pd, and Mf scale interaction with the rest of the MMPI-2 score. This difference was especially obvious in the D and Hy Scale. A statistical test (23) was used to test the correlation matrix between healthy participants and patients with schizophrenia. The result indicated that the MMPI-2 score cor-relations differed between healthy participants and patients with schizophrenia (*p* < 0.001). We have listed all pairs of MMPI-2 clinical scales which the correlation difference between normal and schizophrenia. Pt-D and Pt-Hy have 0.5 correlation differences, and Sc-D, Sc-Hy, and Pa-Hy have 0.4 correlation differences. Healthy individuals have high positive correlations with these pairs but patients with schizophrenia do not. These correlation differences facilitated the Bayesian network to capture the difference between patients with schizophrenia and healthy individuals.

### Scale 278 in the Bayesian network

The Bayesian network facilitates researchers to generate the samples from the probabilistic distribution. The Bayesian network could generate new samples by controlling part of the MMPI-2 clinical scales, so we could conduct a What-if analysis of the MMPI-2 clinical scales and observe the patterns for the rest of the clinical scales. Each time 1,000 random samples of Scale 2, Scale 7, or Scale 8 was generated using schizophrenia patients' demography. Afterward, these random samples were used to generate the rest of the two scales in Scale 2, Scale 7, and Scale 8 with healthy people's network and schizophrenia patients' networks. Table 3 showed the imputed result. All the imputed cells showed statistical differences using a paired-student *t*-test with *p*-values less than 0.001.

**Table 3:** Difference (normal-schizophrenia) in the imputed MMPI-2 scale in schizophrenia patients and healthy people (Diagonal line shows the mean and standard deviation of randomly generated samples).

**Randomly generated sample difference between imputed MMPI-2 scales**			
	Scale 2	Scale 7	Scale 8
Scale 2	53.54 (10.47)	2.65 (0.11)	−2.69 (0.04)
Scale 7	−0.38 (0.12)	53.82 (9.93)	−4.72 (0.05)
Scale 8	3.15 (0.10)	5.25 (0.11)	57.97 (11.81)

## Discussion

The results of this investigation were generally consistent with the results from previous studies, and some innovative discoveries have been explored. F-Sc arc found in this study has been identified in previous studies (19). Si-D and Sc-Pt are the arcs related to the Scale 278 used in differentiating schizophrenia patients. L and K scales were the validity score that quantified a person's behavior on under-reporting their psychological symptoms (18). This evidence showed that the Bayesian network was able to align with the previous findings. In addition, some new findings were obtained through the Bayesian network. We constructed separated Bayesian networks of healthy participants and schizophrenia patients in the MMPI clinical scale and identified all of the arcs, and these arcs represent the correlations between nodes. This method is a generative model which can predict not only schizophrenia but also the range of scores from one scale to another scale.

### Network structure

The main findings of this study were obtained through the network structure on the MMPI clinical scales of healthy participants and patients with schizophrenia. We could observe the correlation and differences between the scales in the arcs of two network structures. We found some arcs in both networks, suggesting that healthy participants and patients with schizophrenia had some common responses on the MMPI clinical scales. Besides, study found differences in scale correlation between the two network structures. The method has identified that the correlation differences between healthy participants and patients with schizophrenia are majorly in Hs, D, Pd, and Mf scales. In the MMPI, these subscales mainly measure abnormal concern for bodily functions (Hs), content related to depression, apathy, pessimism, and slowness of thought and action (D), personality (Pd), and degree of masculinity or femininity (Mf). In addition to these sub-scales related to schizophrenia symptoms, disorganized thinking, emotional indifference, and bizarre behavior as measured by the Sc scale are also important considerations in distinguishing schizophrenia. Furthermore, some arcs existed only in the network structure of healthy participants or patients with schizophrenia. Eight arcs were found only in the network of patients with schizophrenia, but the correlations these arcs were weak. Therefore, it is more worthy of our attention that the Bayesian network method finds that D-Pt, Pd-Ma, and Hs-F arcs only exist in the network structure of healthy participants, and there was a high correlation between these scales.

This study further explored the reasons behind this result and its clinical significance. The difference in the MMPI-2 correlation matrix in network structure could be viewed as the correlation difference between schizophrenia patients' MMPI response and normal people's MMPI response. These differences can be two folds. 1. The structural difference between the two networks indicated that there was a linear correlation in one type of people, and the other did not exhibit such correlation. 2. Even if a link exists in both networks, the linear relationship might behave differently; thus, we can observe such a difference. Bearing the above concepts, we could observe the following findings. The D-Pt arc and Pd-Ma arc were correlated only in the healthy participant network and presented as arcs. This suggests that there are common patterns in these two pairs of responses that patients with schizophrenia do not exhibit. The presence or absence of these two arcs can be observed to assist clinicians in diagnosing schizophrenia. Although the Hs-F arc was moderately correlated in the network structure of both healthy participants and patients with schizophrenia, the Hs-F arc disappeared due to the presence of the Sc-Hs arc in the network structure of patients with schizophrenia. The reason for this phenomenon is that the subject is schizophrenia, so the subject will inevitably have significant clinical manifestations on the two scales of schizophrenia (Sc) and psychopathy (Hs). Thus, Sc-Hs arcs appear in the network structure of patients with schizophrenia but not in the network structure of healthy participants. Therefore, for patients with schizophrenia, the Sc scale is directly related to the Hs scale, which is expressed in the Sc-Hs arc. For healthy participants without Sc-Hs arcs, the association between the Sc scale and Hs scale was indirectly linked through the F scale. Based on the above findings, in clinical practice, when clinical workers try to explain the corresponding clinical symptoms through the correlation between the two scales, the results of the F scale can better explain the results of the Hs scale for healthy individuals, while for patients with schizophrenia, the results of Sc scale can better explain the results of Hs scale.

### The interaction between Scale 278

The other finding of this study was an interaction between Scale 278. Based on previous studies that lower 2, lower 7, and higher 8 are a sign of schizophrenia, we observed that the lower Scale 7 is the consequence of the higher Scale 8 and lower Scale 2. We could observe that setting either Scale 2 at a lower level or Scale 8 at a higher level will result in the conclusion that schizophrenia has a higher Scale 8 and lower Scale 2 and 7. Nonetheless, setting scale 7 at the lower level does not result. Given the above observation, we concluded that the interaction on Scale 278 for schizophrenia was mainly from the higher Scale 8 and lower Scale 2. This result helped us further validate the typical sign of schizophrenia in MPPI-2 and more vividly delineate the relationship between 278 scales. The proposed method provides a novel perspective of interpretation of the interaction between Depression, Psychasthenia, and Schizophrenia scales. This perspective facilitates clinical workers to interpret the meaning behind the scores of the three scales and helps them better understand schizophrenia.

### The Bayesian network

This study also found the unique application value of the Bayesian network in MMPI. The Bayesian network method constructed a network structure for the response patterns of healthy participants and patients with schizophrenia on the MPPI-2 clinical scale, respectively. The Bayesian network structure presented the different degrees of correlation and interactions between each clinical scale. In addition to classifying schizophrenia patients from healthy, researchers can use the Bayesian network to predict the score range of one scale through the score of another scale and infer the overall data from part of the observed data. Therefore, this Bayesian network method can estimate the overall score only by the scores of several scales, which can help clinical workers perform preliminary filtering of schizophrenia more efficiently, lighten the burden of work and improve efficiency.

### Limitations

There are two limitations of this study. One limitation is that the proposed method relied on the nature that the predictors are continuous (MMPI-2 scales), and the target outcome is a binary variable (Diagnosis). Instead of solving a Bayesian network with hybrid variables, separated continuous Bayesian networks for healthy participants and patients with schizophrenia were built. The classification decision was made according to the posterior probability of all MMPI-2 clinical scales. Advance algorithms might be developed to diagnose through the Bayesian networks in the future directly in the mixed variable situation. This is especially valuable for the reason that binary or nominal demographic variables (such as gender) could be included in the model. The other limitation is that the participants of this study came from a single hospital. The demographics might not reflect other cultural and endemic differences in other countries.We would like to expand the population to include more participants from various backgrounds and mental diseases to study the endemic and cultural differences and differential diagnoses between different mental diseases.

## Conclusion

In this study, a separated Bayesian network was used to construct the network structure of the MPPI-2 clinical scale between healthy individuals and patients with schizophrenia. This study investigated the correlation and its differences in the network structure of MMPI-2 MMPI-2 clinical scales between healthy individuals and patients with schizophrenia, Scale 278 of schizophrenia was found to have an interactive relationship, and the results of were due to the interaction between Scale 2 and Scale 8. These findings contribute to a more comprehensive understanding of the clinical symptoms of schizophrenia and provide a new perspective for the clinical diagnosis of medical personnel.

## Data availability statement

The original contributions presented in the study are included in the article/supplementary material, further inquiries can be directed to the corresponding author.

## Ethics statement

The studies involving human participants were reviewed and approved by Institutional Review Board of Wuhan Mental Health Center. Written informed consent to participate in this study was provided by the participants' legal guardian/next of kin.

## Author contributions

YH conducted the analysis and composed the manuscripts. YJ, LD, and WL were responsible for the data, performed experiments, and contributed to the discussion. K-LT, QZ, and PY contributed to the review, and interpretation of study's data. All authors contributed to the article and approved the submitted version.

## Funding

This study was supported by the National Natural Science Foundation of China (No. 71972164).

## Conflict of interest

The authors declare that the research was conducted in the absence of any commercial or financial relationships that could be construed as a potential conflict of interest.

## Publisher's note

All claims expressed in this article are solely those of the authors and do not necessarily represent those of their affiliated organizations, or those of the publisher, the editors and the reviewers. Any product that may be evaluated in this article, or claim that may be made by its manufacturer, is not guaranteed or endorsed by the publisher.
